# Combined Application of Tacrolimus with Cyproconazole, Hymexazol and Novel {2-(3-R-1*H*-1,2,4-triazol-5-yl)phenyl}amines as Antifungals: *In Vitro* Growth Inhibition and *In Silico* Molecular Docking Analysis to Fungal Chitin Deacetylase

**DOI:** 10.3390/jof9010079

**Published:** 2023-01-05

**Authors:** Lyudmyla Antypenko, Fatuma Meyer, Zhanar Sadyk, Konstyantyn Shabelnyk, Sergiy Kovalenko, Karl Gustav Steffens, Leif-Alexander Garbe

**Affiliations:** 1 Faculty of Agriculture and Food Science, Neubrandenburg University of Applied Sciences, Brodaer Str. 2, 17033 Neubrandenburg, Germany; 2 Faculty of Applied Natural Sciences, TH Köln-University of Applied Sciences, Campusplatz 1, 51379 Leverkusen, Germany; 3 Pharmaceutical Chemistry, Organic and Bioorganic Chemistry Department, Zaporizhzhia State Medical University, Mayakovs’ky Ave. 26, 69035 Zaporizhzhia, Ukraine; 4 ZELT–Center for Nutrition and Food Technology, Seestrasse 7A, 17033 Neubrandenburg, Germany

**Keywords:** tacrolimus, synergism, hymexazol, cyproconazole, {2-(3-R-1*H*-1,2,4-triazol-5-yl)phenyl}amines, *A. niger*, *C. higginsianum*, F. oxysporum, P. infestans, chitin deacetylase

## Abstract

Agents with antifungal activity play a vital role as therapeutics in health care, as do fungicides in agriculture. Effectiveness, toxicological profile, and eco-friendliness are among the properties used to select suitable substances. Furthermore, a steady supply of new agents with different modes of action is required to counter the well-known potential of human and phyto-pathogenic fungi to develop resistance against established antifungals. Here, we use an *in vitro* growth assay to investigate the activity of the calcineurin inhibitor tacrolimus in combination with the commercial fungicides cyproconazole and hymexazol, as well as with two earlier reported novel {2-(3-R-1*H*-1,2,4-triazol-5-yl)phenyl}amines, against the fungi *Aspergillus niger*, *Colletotrichum higginsianum*, *Fusarium oxysporum* and the oomycete *Phytophthora infestans*, which are notoriously harmful in agriculture. When tacrolimus was added in a concentration range from 0.25 to 25 mg/L to the tested antifungals (at a fixed concentration of 25 or 50 mg/L), the inhibitory activities were distinctly enhanced. Molecular docking calculations revealed triazole derivative **5**, (2-(3-adamantan-1-yl)-1*H*-1,2,4-triazol-5-yl)-4-chloroaniline), as a potent inhibitor of chitin deacetylases (CDA) of *Aspergillus nidulans* and *A. niger* (*An*CDA and *Ang*CDA, respectively), which was stronger than the previously reported polyoxorin D, J075-4187, and chitotriose. The results are discussed in the context of potential synergism and molecular mode of action.

## 1. Introduction

Global food security primarily relies on the availability of staple food plants such as corn, wheat, rice and potatoes [1]. The steady and sustained supply of these commodities to feed a growing world population is threatened by climate change [2], global resource mismanagement [3] and political conflicts [4]. Furthermore, in growing fields, insect pests, weeds and fungi may have a negative impact on the harvest output. Hence, the application of agrochemicals such as pesticides, herbicides, fungicides and fertilizers is a preeminent measure to protect crop plants and to ensure maximum yields [5]. At the same time, their extensive use can have negative impacts on biodiversity [6], water quality [7], and human health [8]. Fungi are a noteworthy threat to human well-being: they are a health burden for humans and animals (e.g., amphibians) due to mycological infections and diseases, and they destroy one-third of the yearly harvest of crops for food production [9]. Hence, the development of antifungal agents as therapeutics or crop protectants is an ongoing challenge. Fungicides for agriculture are a class of chemicals with a wide variety of molecular structures, but all share antifungal activity. To minimize their potential unwanted effects on health and the environment, their use should be restricted by science-based regulations [10]. The emergence of resistance in target organisms is a serious drawback of fungicides. Their prolonged application favors the selection of mutated fungi that tolerate or overcome the effects of fungicide treatment. Generally, resistance comes with a certain fitness penalty, the extent of which depends on the mode of resistance, as on external factors such as temperature, growth status or humidity [11]. Therefore, an effective use of fungicides should take into account the physiological base of resistance and the onsite regime of application as part of sustainable resistance management. In this context, the type of fungicide and its frequency of application is of eminent importance [12,13].

**Figure 1:**
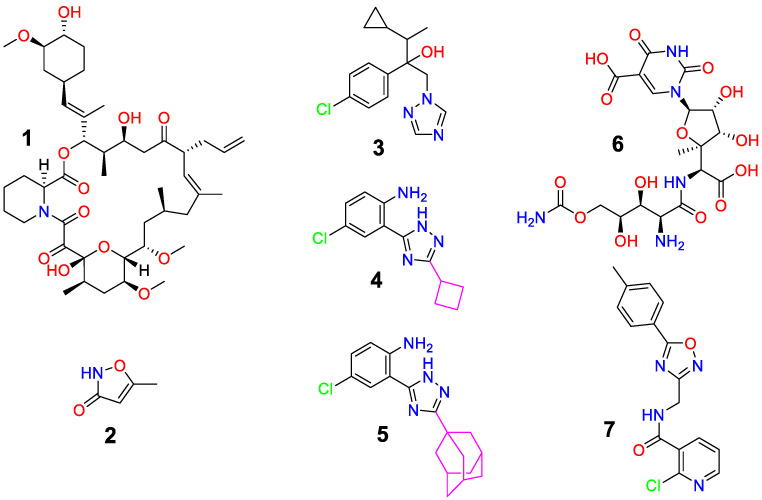
Structures of known antifungal compounds: tacrolimus (**1**), hymexazol (**2**), cyproconazole (**3**), 4-chloro-2-(3-cyclobutyl-1*H*-1,2,4-triazol-5-yl)aniline (**4**), 2-(3-adamantan-1-yl)-1*H*-1,2,4-triazol-5-yl)-4-chloroaniline (**5**), chitin deacetylase inhibitors polyoxorin D (**6**) and J075-4187 (**7**).

In recent *in vitro* studies, we analyzed the antifungal activity of novel triazoles [14] (Figure 1) against a range of fungi of agricultural importance. For almost half a century, azoles have played a preeminent role in effectively managing plant pathogens for the production of major crops such as cereals (*Triticum aestivum, Hordeum vulgare*), oilseed rape (*Brassica napus*), sugar beet (*Beta vulgaris*), bananas (*Musa* spp.), rice (*Oryza sativa*: Asian rice or *Oryza glaberrima*: African rice), soybean (*Glycine max*), oranges (*Citrus* spp.), and turfgrass, minimizing losses worldwide. More than 25 different azole substances developed for crop protection share 20–25% of the value of the fungicide world market [15]. Correspondingly, they are currently detected at considerably high concentrations in surface waters and wastewater in regions of intense agriculture [16]. Furthermore, Berger et al. strongly suggested that recent fungal resistance in medical treatment started *via* an environmental route through the wide exposure of azole fungicides in agriculture [17]. Modification, combination, and repurposing of current antifungals could contribute to managing these problems. Azoles belong to the class of ergosterol biosynthesis inhibitors. They inhibit the specific cytochrome P-450 (CYP) isoform of lanosterol 14-α-demethylase, which catalyzes the formation of ergosterol from lanosterol. Ergosterol is an essential constituent of fungal cell membranes. Azoles can act in the following ways: as a substrate analog *via* hydrophobic interactions in the binding cavity of the enzyme and by strongly coordinating the heme-iron of the active site, thereby blocking the binding of molecular oxygen and interrupting the CYP catalytic cycle. Furthermore, azoles can also affect the biotransformation and bioaccumulation of other chemicals simultaneously administered with them by inhibiting CYP-catalyzed reactions [18]. Recently, we showed in an *in vitro* test the fungicidal activity of ten novel {2-(3-R)-1*H*-1,2,4-triazol-5-yl]phenyl}amines along with the reference fungicides hymexazol and cyproconazole, which also have azole heterocyclic structures, against 12 fungal strains and oomycetes at a concentration of 50 mg/L; additionally, their nonmutagenicity was demonstrated with the reverse *Salmonella* mutagenicity assay (Ames test) [14].

Cyproconazole (Ref: SAN 619, Figure 1) is a strong typical ergosterol biosynthesis inhibitor [19,20] used in a broad spectrum of fungicides mainly applied to protect cereals, beans, asparagus, oilseed rape, sugar beet, apples, almonds, and other field crops against *Septoria*, rust, powdery mildew net blotch, rusts, eyespot, glume blotch, etc. It is moderately toxic to mammals and most aquatic organisms, earthworms and honeybees but highly toxic to birds. Hymexazol (Ref: F 319, Figure 1) is also an effective but less toxic broad-spectrum fungicide recommended for use against gray mold, late blight*, Fusarium* wilt, damping-off, anthracnose in vegetables, rice, cotton, fruit, tobacco crops, soybean, etc. [21,22,23,24]. It can be absorbed directly by plant roots, transferred quickly to multiple parts of the plant, and then transformed into two glucosides (*O*-glucoside and *N*-glucoside). *O*-glucoside can interfere with RNA and DNA synthesis, while *N*-glucoside is associated with plant growth-promoting effects, such as stimulation of lateral root hair development [21,25,26]. Additionally, it is known to inhibit the spore germination of pathogens by combining aluminum and iron ions in the soil under acidic conditions [27].

Recently, the potent *in vitro* antifungal activity of tacrolimus (FK 506 Fujimycin; Figure 1) toward fungi and oomycetes was demonstrated [28]. Tacrolimus is a macrolide lactone isolated and characterized from *Streptomyces tsukubaensis* [29]. As cyclosporin A, it is used as an immunosuppressive agent for post transplantation prophylaxis against organ rejection by interfering with the calcineurin signaling pathway in T cells. At the same time, calcineurin blocking activity protects patients against aggressive pathogenic fungi such as *Aspergillus fumigatus* or *Candida albicans*. Calcineurin is a Ca^2+^-calmodulin-activated phosphatase that regulates fungal physiology, including cell cycle progression, morphogenesis, mating and cytokinesis, recovery from pheromone arrest, cation homeostasis, cell wall biosynthesis, antifungal drug resistance, and virulence [30]. Some components of the calcium-calcineurin signaling pathway vital for fungal growth have been identified as potential and effective targets for the development of new therapeutic drugs [31,32]. Tacrolimus has not been analyzed in the context of plant protection in agriculture thus far, beyond our own study [28], and it is not found in the “Pesticide Properties DataBase” [33].

Aiming to overcome drug resistance, multitarget strategies have attracted attention. Drug combinations have been proven to be a valid and pragmatic concept to design more effective and diverse strategies to deal with harmful and drug-adaptive fungi [34,35]. Interestingly, a synergistic effect of tacrolimus or macrolide everolimus with azoles against *Scedosporium* and *Lomentospora* species, which often cause chronic infection in immunocompromised humans, was observed in *in vitro* and *in vivo* studies [36]. Here, we investigate the potential of tacrolimus in combination with substances of agricultural importance (cyproconazole [19] and hymexazol [21]) as well as two novel triazoles, 4-chloro-2-(3-cyclobutyl-1*H*-1,2,4-triazol-5-yl)aniline (**4**) and 2-(3-adamantan-1-yl)-1*H*-1,2,4-triazol-5-yl)-4-chloroaniline (**5**) (Figure 1), to enhance antifungal activity, to choose more eco-friendly substances and to characterize the impact of dosage on inhibition.

Furthermore, we correlate the activity with the chemical structure. To this end, molecular docking studies were performed while considering chitin deacetylases (CDAs) as the most promising targets. They were discovered from extracts of the fungus *Mucor rouxii* and were further associated with cell wall synthesis by catalyzing the removal of acetyl groups from chitinous substrates, generating various chitosans and influencing their patterns of acetylation [37]. These linear copolymers are important components of fungal cell walls and therefore proved to be excellent antifungal targets [38]. To date, inhibitors of this enzyme class have not been extensively studied. Only polyoxorin D (polyoxin D, polyoxorim; Figure 1), which is a member of the class of polyoxins isolated from the soil organism *Streptomyces cacaoi* var. *asoensis*, was registered as an official antifungal agrochemical and chitin synthase inhibitor (EC 2.4.1.16) [39]. In addition, among 3000 small molecular weight substances, Compound J075-4187 (Figure 1) showed the highest inhibitory activity of 83.77% toward *A. niger* CDA (IC_50_ of 4.24 ± 0.16 μM) [40]. Thus, according to their structural similarity to the studied substances, polyoxorin D [41,42] and J075-4187 [40] were chosen as the comparative standards of affinity toward chitin deacetylases *Ang*CDA from *A. niger* (protein data bank (PDB) ID: 7BLY) [43] and *An*CDA from *A. nidulans* (PDB ID: 2Y8U) [44].

## 2. Materials and Methods

### 2.1. Antifungal Studies

Strains of filamentous fungi were obtained from the following sources: *Colletotrichum higginsianum* MAFF 305635, originally isolated in Japan, *via* the Department of Biology, Friedrich-Alexander-Universität (Erlangen, Germany)*; Fusarium oxysporum* 39/1201 St. 9336 from the Technische Universität (Berlin, Germany); and *Aspergillus niger* DSM 246 from DSMZ (Braunschweig, Germany). The oomycete strain *Phytophthora infestans* GL-1 01/14 wild-type strains were kindly donated by Julius Kühn-Institut (Quedlinburg, Germany). Potato dextrose agar (PDA) was purchased from C. Roth (Karlsruhe, Germany). Hymexazol (98%) was obtained from the Prosperity World Store (Hebei, China). Cyproconazole (99%) was obtained from Sigma Aldrich (Germany). Tacrolimus (99%) was purchased from Huaian Ruanke Trade, Ltd. (Huaian, China). Triazole **1** (4-chloro-2-(3-cyclobutyl-1*H*-1,2,4-triazol-5-yl)aniline) and triazole **2** (2-(3-adamantan-1-yl)-1*H*-1,2,4-triazol-5-yl)-4-chloroaniline) were obtained from Zaporizhzhia State Medical University, Ukraine [14]. Strains were cultivated on PDA for 6 days at 25 °C. Spores from each strain were gently harvested with a sterile glass rod from plate surfaces with deionized water. Spore concentration numbers in suspension were determined microscopically and adjusted to 7.5 × 10^6^ UFC/mL. Clear stock solutions of 5 mg/mL were made of 0.050 g of tested substance in 10 mL of sterile dimethyl sulfoxide (DMSO). One milliliter of each stock solution was mixed *in situ* into 99 mL of PDA prior to solidification to obtain a final concentration of 50 mg/L. In the same way, a series of PDA with tested individual or mixed compounds were prepared to achieve final concentrations of 0.25–50 mg/L. Nine milliliters of each mixture was poured into 6 cm diameter petri dishes. After solidification, the central hole (diameter: 2.5 mm) was cut out and inoculated with 6.5 µL spore suspension. Plates were incubated at 25 °C (+/−1 °C) for 6 days. Control plates containing only PDA and deionized water were prepared in the same way. Inhibitory effects (I %) were determined by analyzing growth zone diameters and calculated as I % = [(C − T)/(C − 2.5 mm)]) × 100, where C (mm) represents the growth zone of control PDA + 1% DMSO and T (mm) represents the average growth zone in the presence of reference or test substances [14]. The enhancement or decline of microorganism growth was deducted from the activity of mixtures toward individual inhibition by substances. All growth experiments were carried out in triplicate. Inhibitory effects, means and standard deviations were calculated with Excel 2016 software (Microsoft, USA). Data were measured as the means ± standard errors (SE). Statistically, multiple comparisons of normally distributed data were achieved *via* one-way analysis of variance (ANOVA) using SPSS 26.0 statistical software (SPSS Inc., Chicago, IL, USA) followed by Tukey’s test for post hoc analysis. A p value of ≤0.05 was considered significant. Spearman’s correlation coefficients were calculated by SPSS 26.0.

### 2.2. Molecular Docking Studies

Macromolecules from the Protein Data Bank (PDB) were used as the biological targets, namely, *A. niger* CDA (*Ang*CDA; PDB ID: 7BLY) [43] and *A. nidulans* CDA (*An*CDA; PDB ID: 2Y8U) [44]. Polyoxorin D [42] and J075-4187 (2-chloro-*N*-((5-(*p*-tolyl)-1,2,4-oxadiazol-3-yl)methyl)nicotinamide) [40] were chosen as the references. The seven mol files of tacrolimus (**1**), hymexazol (**2**), cyproconazole (**3**), triazoles (**4)**, and (**5)**, polyoxorin D (**6**), J075-4187 (**7**) (Figure 1) were drawn by ChemDraw Professional 15.0 and optimized by HyperChem 8.0.8; mol files were converted to pdb by Open Babel GUI 2.3.2; pdb files were converted to pdbqt by AutoDocTools 1.5.6. Vina 1.1.2 was used to carry out docking studies [45]. The following grid boxes were used: for PDB ID: 7BLY, center_x = 23.501; center_y = 53.533; center_z = -21.063; size_x = 22; size_y = 22; size_z = 22; for PDB ID: 2Y8U, center_x = -0.565; center_y = -38.599; center_z = 25.537; size_x = 22; size_y = 22; size_z = 22. Discovery Studio v17.2.0.16349 was used for visualization. To validate the docking method by the value of RMSD (root-mean-squared deviation), which characterizes the degree of reliable docking probability, the reference ligands were extracted and then reused for the redocking process [46]. If the found pose has an RMSD less than 2 Å relative to the X-ray conformation, then it is generally considered a docking success [47]. RSMD values between the experimental and the reference conformation ligands were calculated to be 1.001 Å for *An*CDA and 0.664 Å for *Ang*CDA *via* DockRMSD available online [48]. Therefore, the study is considered reliable.

## 3. Results and Discussion

### 3.1. Antifungal Studies

Experiments were designed to reveal potential additive or synergistic effects when combining a concentration range of tacrolimus (**1**: 0.25–25 mg/L) with a fixed concentration of novel triazole (**4**: 50 mg/L; **5**: 25 mg/L) or reference antifungals such as hymexazol (**2**; 50 mg/L) and cyproconazole (**3**: 25 mg/L). Among the described novel antifungal triazoles [14] for this study, 4-chloro-2-(3-cyclobutyl-1*H*-1,2,4-triazol-5-yl)aniline (**4**) and 2-(3-adamantan-1-yl)-1*H*-1,2,4-triazol-5-yl)-4-chloroaniline (**5**), with mean antifungal activities of 38.7% and 56.8% at 50 mg/L, respectively, were selected. Test strains *A. niger*, *C. higginsianum*, *F. oxysporum*, and *P. infestans GC-1* were chosen since in our previous study [14] they exhibited low sensitivity toward eco-friendly hymexazol. It is further noteworthy that *Colletotrichum* spp. and *F. oxysporum* were included in the list of the 10 most important plant pathogenic fungi worldwide [49]. Infection by *Colletotrichum* spp. can result in several diseases, including anthracnose, fruiting after flowering and postharvest anthracnose [50], and diseases of lime [51,52], grapefruit [53] and pomegranate [54]. Aspergilli comprise a large and diverse genus (approximately 180 species) of filamentous fungi, including several well-known species with substantial commercial value (*A. oryzae* and *A. niger*), pathogenic potential (*A. parasiticus* and *A. fumigatus*) and toxin-producing contaminants of food and feed (*A. flavus*) [55]. *F. oxysporum* belongs to a class of filamentous fungi that includes endophytes, saprophytes, and pathogens causing vascular wilt disease, damping-off, and crown or root rots [56,57]. *P. infestans* is a hemibiotrophic oomycete pathogen that confers *late blight*, one of the most devastating plant diseases worldwide, with high aggressiveness and marked host adaptability to potato [58] and tomato [59]. It is effectively spread by infected vegetative material.

**Figure 2:**
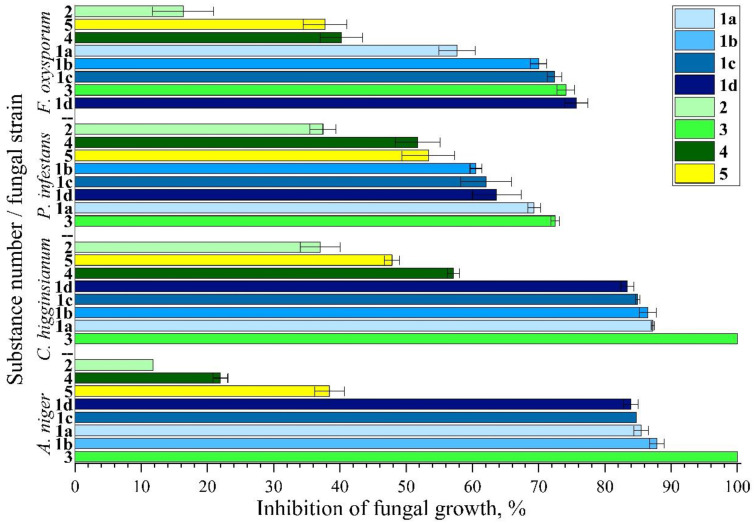
Inhibition rates of the substances cyproconazole (**3**) and triazole (**4**) at 25 mg/L, respectively; hymexazol (**2**) and triazole (**5**) at 50 mg/L, respectively; tacrolimus (**1a**: 0.25 mg/L, **1b**: 1 mg/L, **1c**: 10 mg/L, **1d**: 25 mg/L) against *P. infestans*, *F. oxysporum*, *C. higginsianum*, and *A. niger*. Experiments were carried out in triplicate (error bars: standard deviation (SD).

The basal antifungal activities are shown for all substances in Figure 2 Supplementary Material Tables S1–S4).

If the minimum inhibitory concentration (MIC) is considered 50% inhibition [60], then it was observed for tacrolimus at all concentrations applied (**1a**–**1d**). With the exception of *F. oxysporum,* no distinct correlation of inhibition and concentration range (0.25–25 mg/L) was found. Overall, tacrolimus was less effective toward *F. oxysporum* and *P infestans*. The activity of the reference antifungal hymexazol (**2**) at 50 mg/L was lower than 50% for all strains. On the other hand, cyproconazole (**3**) at 25 mg/L conferred 100% inhibition toward *A. niger* and *C. higginsianum*, whereas *F. oxysporum* and *P. infestans* were more resistant (80% inhibition).

**Figure 3:**
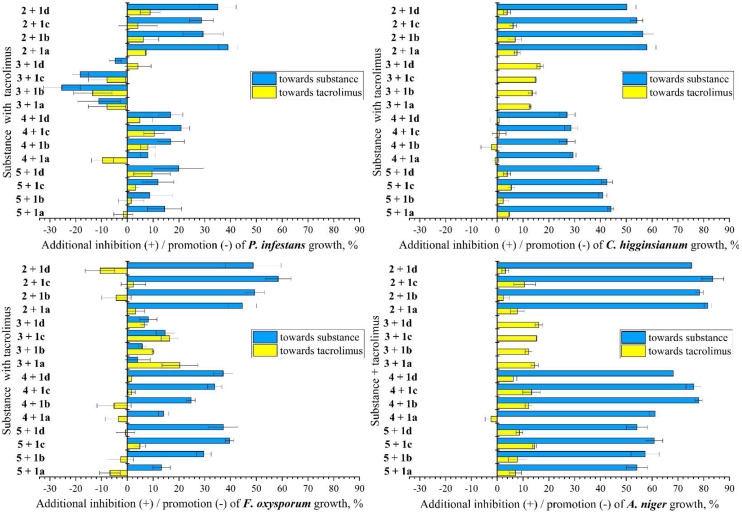
The additional growth inhibition (+)/promotion (−) of *P. infestans*, *C. higginsianum*, *F. oxysporum*, and *A.niger* was found, when tacrolimus (**1a**: 0.25 mg/L; **1b**: 1 mg/L; **1c**: 10 mg/L; **1d**: 25 mg/L) was combined with hymexazol (**2**): 50 mg/L; cyproconazole (**3**): 25 mg/L; triazoles (**4**), and (**5**): 50 mg/L. Experiments were carried out in triplicate (error bars: standard deviation).

Novel triazoles: 4-chloro-2-(3-cyclobutyl-1*H*-1,2,4-triazol-5-yl)aniline (**4**) at 25 mg/L and 2-(3-adamantan-1-yl)-1*H*-1,2,4-triazol-5-yl)-4-chloroaniline (**5**) at 50 mg/L showed intermediate activity at (*C. higginsianum*; *P. infestans*) or below MIC (*F. oxysporum*; *A. niger*). Generally, *F. oxysporum* and *P. infestans* were less sensitive to applied antifungals. Especially for the latter, the peculiarities of fungal wall structure and/or their metabolism, as well as its high mutation rate, may confer its elevated resistance [59]. Interestingly, *A. niger* showed a high sensitivity toward tacrolimus and cyproconazole but was quite resistant against hymexazol and triazoles. Based on these nonuniform findings, experiments with combinations of antifungals and tacrolimus were carried out (Figure 3, Supplementary Material Tables S5–S8).

For all strains in the presence of tacrolimus (**1**) and hymexazol (**2**), the inhibition rate was enhanced when compared with hymexazol alone. This additive effect was greatest with *A. niger*, which showed an exceptionally low sensitivity against hymexazol (Figure 2). Compared to the results of tacrolimus alone, the combination with hymexazol only gave slightly higher inhibition rates; for *F. oxysporum,* the combination of these antifungals even reduced the inhibition that was observed with tacrolimus alone. Application of cyproconazole (**3**) with tacrolimus exhibited a medium enhancement with *F. oxysporum* when compared to each substance alone. Unexpectedly, a marked growth promotion of this combination was revealed for *P. infestans*. Here, the special physiological properties of this oomycete (s. above) might be the cause. For *C. higginsianum* and *A. niger,* an enhancement of inhibition could only be observed toward tacrolimus, as cyproconazole (25 mg/L) alone already conferred 100% inhibition.

Triazoles **4** and **5** showed medium additional inhibition rates in combination with tacrolimus. These effects were most distinct when applying **4** and **5** together with tacrolimus at 10 mg/L (**1c**) to *A. niger*. An opposite impact on tacrolimus growth inhibition at the lower concentrations (0.25 mg/L; 1 mg/L) was observed in combination with triazole **4** against *C. higginsianum* (−0.78%; −2.28%), *F. oxysporum* (−3.44%; −5.13%), *A. niger* (−2.36%), and *P. infestans* (−9.61%). These findings may reflect a hormesis effect, i.e. a stimulation of response at low doses and inhibition of response at high doses [61,62]. Here, it can be caused by the induction of specific CYP isoforms that catalyze oxidative biotransformation reactions of active substances [63]. An additional growth inhibition correlating with the tacrolimus concentration range (0.25–25 mg/L) was noted for *F. oxysporum* in combination with triazoles **4** or **5**. Notably, a strong significant (*p* = 0.010) Spearman’s correlation coefficient of ρ = 1.000 between tacrolimus concentration and inhibition level of triazole **4** against *F. oxysporum* was calculated. Correspondingly, it was calculated for triazole **5** at ρ = 0.900 (*p* = 0.037). Considering the physico-chemical parameters of the studied triazoles, the number of rotational bonds conferred the highest influence on the antifungal activities, with a strong Spearman’s rho of 0.872, significant at the level of *p* =.054 (Supplementary Material Table S9) [64].

### 3.2. Molecular Docking

Molecular docking is a new *in silico* tool to predict molecular interactions, which do not depend on physicochemical molecular descriptors but rely on a minimum amount of information from mathematical topological models and their physicochemical interpretations. Its application successfully led to the design of new active lead compounds for biological and pharmaceutical purposes [65].

Previously, possible antifungal activity mechanisms against six common targets, sterol 14α-demethylase (CYP51), *N*-myristoyltransferase (NMT), UDP-*N*-acetylmuramoyl-L-alanine: D-glutamate ligase (MurD), topoisomerase II (TopoII), L-glutamine: D-fructose-6-phosphate amidotransferase (GlcN-6-P), and secreted aspartic proteinase (SAP2), were analyzed by applying *in silico* molecular docking studies [14,19]. It was shown that tacrolimus had the highest affinity (−9.8 kcal/mol) to the secreted aspartic proteinase (SAP2); as expected, the affinities of novel triazoles (**4**, **5**) and cyproconazole scored highest toward 14α-demethylase (CYP51). Hymexazol exhibited the highest affinity toward *N*-myristoyltransferase (NMT). Here, chitin deacetylase (CDA) is analyzed as an antifungal target.

**Figure 4:**
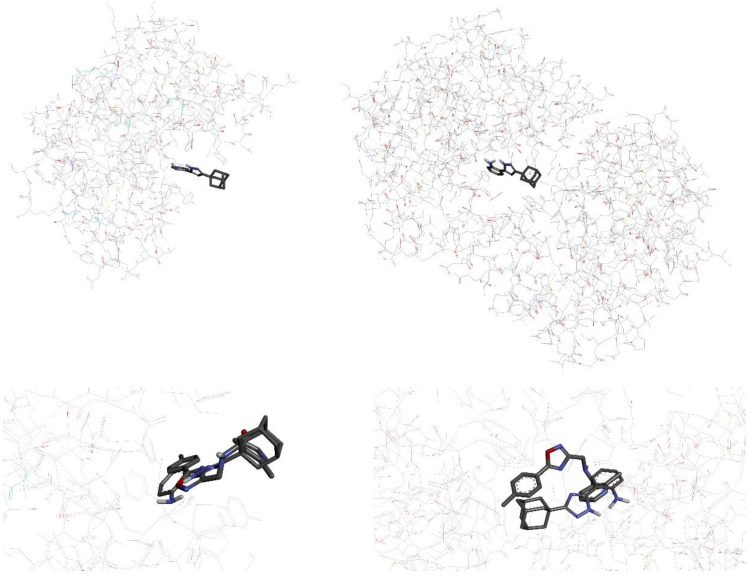
Visual representation (3D) of triazole **5** fit into the active site of *A. niger* chitin deacetylase (*Ang*CDA; left up) and *A. nidulans* chitin deacetylase (*An*CDA; right up); configuration of triazole **5** simultaneously fitting with J0750-4187 into the CDA-binding pocket of *A. niger* (left down) and *A. nidulans* (right down).

As mentioned by Bonin et al., the fungal CDAs of *A. niger* and *A. nidulans* have open active sites, leaving them accessible for antifungals [37]. Correspondingly, we performed molecular docking studies on CDA from these fungi (*Ang*CDA; PDB ID: 7BLY [43]; *An*CDA; PDB ID: 2Y8U [44]) to characterize the affinities of the studied substances. As shown in Figure 4, both comparative standards J075-4187 (**7**) and triazole **5** fit nicely into the active site of the proteins.

Whereas in *Ang*CDA their spatial position is almost the same, they differ in *An*CDA due to the flexibility of their skeleton. In this respect, the 3D pictures of the same enzymes (CDA) of *Aspergillus* species reveal subtle differences within the structure, which have a marked influence on the binding affinities of the studied substances.

The calculated affinity scores of all substances considered here are shown in Table 1.

The triazole **5** affinity score was highest toward *Ang*CDA (−8.2 kcal/mol) and toward *An*CDA (−9.6 kcal/mol) (Table 1). Notably, it was higher than those calculated for polyoxorin D (−7.1 kcal/mol) or reported chitotriose (−6.1 kcal/mol) and ethylenediamine tetraacetic acid (EDTA; −6.8 kcal/mol), which also proved to decrease CDA enzyme activity *in vitro* [66,67]. The calculated affinity for triazole **4** to both *Ang*CDA and *An*CDA was inferior only to triazole **5** and J075-4187. All other analyzed compounds had moderate affinities toward both enzymes, with hymexazol as the weakest substance.

**Figure 5:**
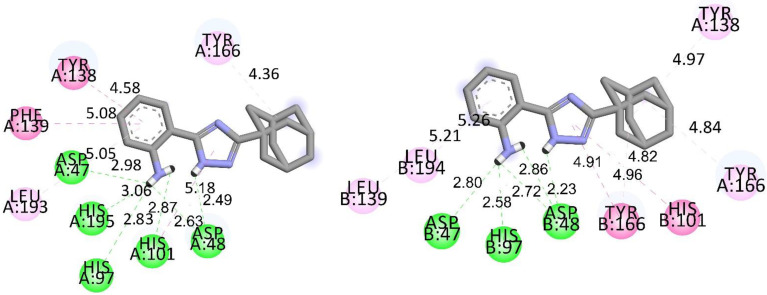
Calculated bonds and interactions (2D) of triazole **5** toward amino acid residues in CDA active sites of *A. niger* (*Ang*CDA; left) and of *A. nidulans* (*An*CDA; right); bond distances indicated in Å. Green: Conventional Hydrogen Bond; magenta: π-π T-shaped/Amide-π Stacked/π-π Stacked; pink: π-Alkyl (Tables S10 and S11, Supplementary Material).

The active site of *An*CDA, and in a similar configuration to *AngC*DA, includes the HIS-HIS-ASP metal-binding triad (HIS97, HIS101, and ASP48), a catalytic acid (HIS196, aiding sugar departure), and a catalytic acid (ASP47) [40]. CDAs of *Colletotrichum sp.* [67] and *A. niger* [40], certain carboxylic acids, such as EDTA, ultimately fit into this structure. Therefore, EDTA binds to *Ang*CDA *via* four hydrogen bonds that involve HIS97, TYR138, TYR166, and HIS199. Correspondingly, with the abovementioned amino acids, the formation of six hydrogen bonds for *Ang*CDA and five for *An*CDA for triazole **5** (Figure 5) was calculated. For triazole **4,** the formation of seven (*Ang*CDA) and five (*An*CDA) hydrogen bonds was found (Supplementary Material Tables S10 and S11). Considering reference substance **7**, no hydrogen bonds were formed with *Ang*CDA, but five hydrogen bonds were formed with *An*CDA.

For the example of substance **5,** the observed differences toward CDA from two PDB IDs can be shown (Figure 5, Supplementary Material Tables S10 and S11). On average, all bonds calculated for *Ang*CDA were slightly shorter than those for *An*CDA. The distance difference was highest at approximately 0.5 Å for TYR138 and TYR166. In the case of *An*CDA, one additional conventional hydrogen bond formed with ASP48; for *Ang*CDA, two bonds formed with HIS101 and HIS195. Only for *Ang*CDA were hydrophobic π-π T-shaped bonds to HIS195 and PHE139 observed, along with an additional amide-π stacked bond to PHE139. The bonds with leucine were formed in each case but with different residues: LEU193 for *Ang*CDA and LEU139 and LEU194 for *An*CDA. For *An*CDA, additional hydrophobic π-π stacked and π-alkyl bonds to TYR166 were observed. Thus, the substance affinities on CDAs from the two aspergilli were different. Nevertheless, the order of affinity of all studied substances was the same in each case. The formation and types of all bonds for substances with corresponding distances are given in Supplementary Material Tables S10 and S11.

## 4. Proposed Activity Mechanisms and Future Perspectives

A concise interpretation of results from growth experiments and docking studies is this: the novel triazoles **4**, **5** and cyproconazole disrupt wall synthesis by interfering with enzyme activities of CYP [14] or/and CDA, allowing tacrolimus to enter the cell and inhibit the calcineurin pathway. Furthermore, as tacrolimus has been shown to be an inhibitor of the multidrug efflux pump P-glycoprotein (P-gp) [68,69], it may increase the intracellular concentration of azoles or other antifungals with concomitant effects on essential cellular processes. Interestingly, sirolimus, a macrolide similar to tacrolimus, was shown to modulate autophagic activity and membrane permeability [70]. In this context, several manners of synergism and antagonism, which were described for fungicide mixtures with azoles [71], should be considered: being a substrate or inhibitor of multidrug transporter P-glycoprotein, activation or inhibition of passive or energy-dependent efflux, and mitochondrial respiratory inhibition. These observations may be reflected by our findings of growth promotion when applying tacrolimus together with further antifungals; this was especially obvious for *P. infestans* GL-1 and *F. oxysporum* in combination with azoles or cyproconazole.

There are additional promising pathways of triazoles or hymexazol in combination with tacrolimus: EDTA was shown to effectively target cucurbit powdery mildew disease, biotrophic ascomycete fungi from the order *Erysiphales*, necrotrophic fungi such as *Botrytis cinerea* (gray mold) and *Penicillium digitatum* (green mold) [66,67]. Since for triazole **5** a higher affinity to *An*CDA as for EDTA was calculated, its application in conjunction with tacrolimus toward these fungi is also promising.

As shown by Thomas et al. [72], the application of mixtures might confer additional cost benefits. For example, in human post transplantation treatment, the cost of sirolimus at 2 mg/day was $625/month compared with $86 for the combination with ketoconazole. In a similar way, economic advantages may result from the combinations of two or more antifungals. Further investigations are needed to determine whether other fungal species are more sensitive toward combinations of hymexazol, novel triazoles and tacrolimus and whether synergistic effects at lower exposure concentrations can be discovered.

Generally, new application regimes of agrochemicals should also be evaluated regarding potentially dangerous actions in human health care: Berger et al. [17] discussed the potential link of the emergence of azole-resistant pathogenic *A. fumigatus* and the use of this fungicide class in agriculture. Therefore, accompanying studies including the determination of absorption, distribution, metabolism, and excretion (ADME parameters) should be included in the development of new substances of agricultural importance [64]. Moreover, the nephrotoxicity of antifungal agents can be reduced with liposome-based and submicronic colloidal systems [73]. The control of drug release by nanosized carriers, e.g., microemulsions, vesicular carriers, nanosuspensions, and wet media milling technology, also has a great impact on minimizing systemic absorption and decreasing toxicity, as in medicine and agriculture [74]. Recently, Liu et al. [75] reported that modified diatomite was a highly efficient and stable carrier of hymexazol to prepare pesticide sustained-release agents suitable for neutral and acidic soil environments. Finally, possibly deleterious effects on the environment by the extensive use of combinations of active agents should be ruled out by appropriate toxicity studies [26].

## 5. Conclusions

The antifungal activity of the known antifungal hymexazol was increased by approximately 2–8 times in combination with only 0.25 μg/mL tacrolimus against the studied fungi *A. niger*, *C. higginsianum*, *F. oxysporum*, and the oomycete *P. infestans*. Moreover, the novel triazole **5**, (2-(3-adamantan-1-yl)-1*H*-1,2,4-triazol-5-yl)-4-chloroaniline), at a concentration two times lower (25 μg/mL) than the reference hymexazol after the addition of tacrolimus at different dosages (0.25–25 μg/mL), was more active against *A. niger* and *F. oxysporum,* practically achieving the results of cyproconazole. Therefore, tacrolimus addition can make preferable usage of eco-friendly and economically efficient hymexazol over the more toxic cyproconazole, and novel potent antifungal agents are presented. In addition, the effective dosages of the substances may be even further decreased upon additional investigation. According to the calculated molecular docking, triazole **5** has a high affinity for *AngCDA* and *AnCDA*, which is stronger than those reported for chitotriose [66], polyoxorin D [41,42], and J075-4187 [40]. An expansion of the concentration ranges, especially for tacrolimus, might reveal additional synergistic activities, which allow the use of combinations of more eco-friendly antifungals at lower concentrations, with the same effectiveness. In addition to a possible delay in the development of resistance, a corresponding application regime may reduce costs and offer benefits to the environment.

## Figures and Tables

**Table 1:** Affinity to binding sites of *Aspergillus niger* CDA (PDB ID: 7BLY) and *A. nidulans* CDA (PDB ID: 2Y8U), kcal/mol.

Substance	7BLY	2Y8U
Triazole (**5**)	−8.2	−9.6
J075-4187 (**7**)	−7.5	−9.0
Triazole (**4**)	−7.5	−7.8
Cyproconazole (**3**)	−6.5	−7.1
Polyoxorin D (**6**)	−6.2	−7.1
Tacrolimus (**1**)	−4.6	−6.9
Hymexazol (**2**)	−5.2	−5.1

## Data Availability

All raw data from this study are available upon reasonable request to the corresponding author.

## Supplementary Materials

The following supporting information can be downloaded at: https://www.mdpi.com/article/10.3390/jof9010079/s1, Tables S1–S4: Antifungal activity (%) against *P. infestans, F. oxysporum, C. higginsianum, and A. niger,* respectively. Tables S5–S8: Additional inhibition/promotion (%) against *P. infestans*, *F. oxysporum*, *C. higginsianum*, and *A. niger*. Table S9: Spearman’s correlations of substances’ **1**–**5** physico-chemical data versus their average individual antifungal activities. Tables S10 and S11*:* Descriptives of formed bonds to chitin deacetylase of *A. niger* (PDB ID: 7BLY) and *A. nidulans* (PDB ID: 2Y8U) in order of decreasing affinity.
