# Supplementary material for: Combined Application of Tacrolimus with Cyproconazole, Hymexazol and Novel {2-(3-R-1H-1,2,4-triazol-5-yl)phenyl}amines as Antifungals: In Vitro Growth Inhibition and In Silico Molecular Docking Analysis to Fungal Chitin Deacetylase
Source: J Fungi (Basel). 2023 Jan 5;9(1):79. doi: 10.3390/jof9010079 (PMC9866229; doi:10.3390/jof9010079)
Supplement: Supplementary file 1 [file jof-09-00079-s001.zip › jof-2121428-supplementary.pdf]

Supplementary Material

Combined application of tacrolimus with cyproconazole, hymexazol and novel {2-(3-R-1*H*-1,2,4-triazol-5-yl)phenyl}amines as antifungals: *In vitro* growth inhibition and *in silico* molecular docking analysis to fungal chitin deacetylase

Lyudmyla Antypenko [1], Fatuma Meyer [1], Zhanar Sadyk [1,2], Konstyantyn Shabelnyk [3], Sergiy Kovalenko [3], Karl Gustav Steffens [1] and Leif-Alexander Garbe [1,4,*]

[1] Faculty of Agriculture and Food Science, Neubrandenburg University of Applied, Brodaer Str. 2, 17033 Neubrandenburg, Germany
[2] Faculty of Applied Natural Sciences, TH Köln-University of Applied Sciences, Campusplatz 1, 51379 Leverkusen, Germany
[3] Pharmaceutical Chemistry, Organic and Bioorganic Chemistry Department, Zaporizhzhia State Medical University, Mayakovs'ky Ave. 26, 69035 Zaporizhzhia, Ukraine
[4] ZELT–Center for Nutrition and Food Technology, Seestrasse 7A, 17033 Neubrandenburg, Germany
*Correspondence: garbe@hs-nb.de; Tel.: +49-395-5693-1004

Abbreviations: tacrolimus (**1**); hymexazol (**2**); cyproconazole (**3**); 4-chloro-2-(3-cyclobutyl-1*H*-1,2,4-triazol-5-yl)aniline (**4**); 2-(3-adamantan-1-yl)-1*H*-1,2,4-triazol-5-yl)-4-chloroaniline (**5**); polyoxorin D (**6**), J075-4187 (2-chloro-*N*-((5-(*p*-tolyl)-1,2,4-oxadiazol-3-yl)methyl)nicotin-amide) (**7**).

Table S1. Antifungal activity against *P. infestans, %* (SD: standard deviation)

Substance (concentration in mg/L)	Diameter of growth zones (mm) and inhibition rate (%)							
	**i**		**ii**		**iii**		**mean**	**SD**
	**(mm)**	**(%)**	**(mm)**	**(%)**	**(mm)**	**(%)**	**(%)**	**(%)**
1d **(25)**	19	59.26	16.0	68.24	18.0	63.53	63.67	3.67
1c **(10)**	20	56.79	18.0	63.53	17.0	65.88	62.07	3.85
1b **(1)**	19	59.26	19.0	61.18	19.0	61.18	60.54	0.90
1a **(0.25)**	15	69.14	15.0	70.59	16.0	68.24	69.32	0.97
2 **(50)**	28.0	37.04	30.0	35.29	28.0	40.00	37.44	1.94
2 **(50)** + 1d **(25)**	14.0	71.60	12.0	77.65	16.0	68.24	72.50	3.89
2 **(50)** + 1c **(10)**	15.0	69.14	17.0	65.88	18.0	63.53	66.18	2.30
2 **(50)** + 1b **(1)**	19.0	59.26	14.0	72.94	16.0	68.24	66.81	5.68
2 **(50)** + 1a **(0.25)**	12.0	76.54	12.0	77.65	13.0	75.29	76.49	0.96
3 **(25)**	14.0	71.60	14.0	72.94	14.0	72.94	72.50	0.63
3 **(25)** + 1d **(25)**	15.0	69.14	16.0	68.24	17.0	65.88	67.75	1.37
3 **(25)** + 1c **(10)**	20.0	56.79	22.0	54.12	23.0	51.76	54.22	2.05
3 **(25)** + 1b **(1)**	21.0	54.32	27.0	42.35	26.0	44.71	47.13	5.18
3 **(25)** + 1a **(0.25)**	15.0	69.14	22.0	54.12	19.0	61.18	61.48	6.13
4 **(50)**	24.0	46.91	22.0	54.12	22.0	54.12	51.72	3.40
4 **(50)** + 1d **(25)**	15.0	69.14	16.0	68.24	16.0	68.24	68.54	0.42
4 **(50)** + 1c **(10)**	14.0	71.60	14.0	72.94	14.0	72.94	72.50	0.63
4 **(50)** + 1b **(1)**	15.0	69.14	15.0	70.59	17.0	65.88	68.54	1.97
4 **(50)** + 1a **(0.25)**	20.0	56.79	20.0	58.82	18.0	63.53	59.71	2.82
5 **(25)**	22.0	51.85	20.0	58.82	24.0	49.41	53.36	3.99
5 **(25)** + 1d **(25)**	15.0	69.14	15.0	70.59	11.0	80.00	73.24	4.82
5 **(25)** + 1c **(10)**	18.0	61.73	17.0	65.88	16.0	68.24	65.28	2.69
5 **(25)** + 1b **(1)**	20.0	56.79	19.0	61.18	16.0	68.24	62.07	4.71
5 **(25)** + 1a **(0.25)**	14.0	71.60	17.0	65.88	17.0	65.88	67.79	2.70
PDA + 1%DMSO	43	0.00	45	0.00	45	0.00	0.00	0.00

Table S2. Antifungal activity against *F. oxysporum, %* (SD: standard deviation)

Substance (concentration in mg/L)	Diameter of growth zones (mm) and inhibition rate (%)							
	**i**		**ii**		**iii**		**mean**	**SD**
	**(mm)**	**(%)**	**(mm)**	**(%)**	**(mm)**	**(%)**	**(%)**	**(%)**
1d **(25)**	12	75.95	13	73.42	12	77.65	75.67	1.74
1c **(10)**	14	70.89	13	73.42	14	72.94	72.41	1.10
1b **(1)**	14	70.89	14	70.89	16	68.24	70.00	1.25
1a **(0.25)**	18	60.76	19	58.23	22	54.12	57.70	2.74
2 **(50)**	38.0	10.13	35.0	17.72	36.0	21.18	16.34	4.62
2 **(50)** + 1d **(25)**	14.0	70.89	17.0	63.29	19.0	61.18	65.12	4.17
2 **(50)** + 1c **(10)**	13.0	73.42	14.0	70.89	11.0	80.00	74.77	3.84
2 **(50)** + 1b **(1)**	17.0	63.29	17.0	63.29	15.0	70.59	65.72	3.44
2 **(50)** + 1a **(0.25)**	18.0	60.76	18.0	60.76	19.0	61.18	60.90	0.20
3 **(25)**	12.0	75.95	13.0	73.42	14.0	72.94	74.10	1.32
3 **(25)** + 1d **(25)**	10.0	81.01	10.0	81.01	9.0	84.71	82.24	1.74
3 **(25)** + 1c **(10)**	7.0	88.61	8.0	86.08	6.0	91.76	88.82	2.33
3 **(25)** + 1b **(1)**	10.0	81.01	10.0	81.01	12.0	77.65	79.89	1.59
3 **(25)** + 1a **(0.25)**	12.0	75.95	12.0	75.95	10.0	82.35	78.08	3.02
4 **(50)**	27.0	37.97	27.0	37.97	26.0	44.71	40.22	3.17
4 **(50)** + 1d **(25)**	11.0	78.48	12.0	75.95	12.0	77.65	77.36	1.05
4 **(50)** + 1c **(10)**	13.0	73.42	13.0	73.42	13.0	75.29	74.04	0.88
4 **(50)** + 1b **(1)**	17.0	63.29	18.0	60.76	15.0	70.59	64.88	4.17
4 **(50)** + 1a **(0.25)**	21.0	53.16	21.0	53.16	21.0	56.47	54.27	1.56
5 **(25)**	28.0	35.44	28.0	35.44	27.0	42.35	37.75	3.26
5 **(25)** + 1d **(25)**	12.0	75.95	12.0	75.95	14.0	72.94	74.95	1.42
5 **(25)** + 1c **(10)**	12.0	75.95	12.0	75.95	11.0	80.00	77.30	1.91
5 **(25)** + 1b **(1)**	15.0	68.35	17.0	63.29	15.0	70.59	67.41	3.05
5 **(25)** + 1a **(0.25)**	22.0	50.63	22.0	50.63	23.0	51.76	51.01	0.53
PDA + 1%DMSO	42	0.00	42	0.00	45	0.00	0.00	0.00

Table S3. Antifungal activity against *C. higginsianum*, % (SD: standard deviation)

Substance (concentration in mg/L)	Diameter of growth zones (mm) and inhibition rate (%)							
	**i**		**ii**		**iii**		**mean**	**SD**
	**(mm)**	**(%)**	**(mm)**	**(%)**	**(mm)**	**(%)**	**(%)**	**(%)**
1d **(25)**	10	82.35	10	83.15	9	84.71	83.40	0.98
1c **(10)**	9	84.71	9	85.39	9	84.71	84.94	0.32
1b **(1)**	8	87.06	8	87.64	9	84.71	86.47	1.27
1a **(0.25)**	8	87.06	8	87.64	8	87.06	87.25	0.27
2 **(50)**	28	40.00	30	38.20	31	32.94	37.05	3.00
2 **(50)** + 1d **(25)**	8	87.06	8	87.64	8	87.06	87.25	0.27
2 **(50)** + 1c **(10)**	6	91.76	6	92.13	7	89.41	91.10	1.21
2 **(50)** + 1b **(1)**	5	94.12	6	92.13	5	94.12	93.46	0.93
2 **(50)** + 1a **(0.25)**	5	94.12	4	96.63	5	94.12	94.95	1.18
3 **(25)**	2.5	100.00	2.5	100.00	2.5	100.00	100.00	0.00
3 **(25)** + 1d **(25)**	2.5	100.00	2.5	100.00	2.5	100.00	100.00	0.00
3 **(25)** + 1c **(10)**	2.5	100.00	2.5	100.00	2.5	100.00	100.00	0.00
3 **(25)** + 1b **(1)**	2.5	100.00	2.5	100.00	2.5	100.00	100.00	0.00
3 **(25)** + 1a **(0.25)**	2.5	100.00	2.5	100.00	2.5	100.00	100.00	0.00
4 **(50)**	21	56.47	21	58.43	21	56.47	57.12	0.92
4 **(50)** + 1d **(25)**	8	87.06	10	83.15	10	82.35	84.19	2.06
4 **(50)** + 1c **(10)**	8	87.06	8	87.64	10	82.35	85.68	2.37
4 **(50)** + 1b **(1)**	10	82.35	10	83.15	8	87.06	84.19	2.06
4 **(50)** + 1a **(0.25)**	8	87.06	8	87.64	9	84.71	86.47	1.27
5 **(25)**	25	47.06	25	49.44	25	47.06	47.85	1.12
5 **(25)** + 1d **(25)**	8	87.06	8	87.64	8	87.06	87.25	0.27
5 **(25)** + 1c **(10)**	7	89.41	7	89.89	6	91.76	90.35	1.02
5 **(25)** + 1b **(1)**	8	87.06	6	92.13	8	87.06	88.75	2.39
5 **(25)** + 1a **(0.25)**	6	91.76	6	92.13	6	91.76	91.89	0.17
PDA + 1%DMSO	45	0	45	0.00	45	0.00	0.00	0.00

Table S4. Antifungal activity against *A. niger, %* (SD: standard deviation)

Substance (concentration in mg/L)	Diameter of growth zones (mm) and inhibition rate (%)							
	**i**		**ii**		**iii**		**mean**	**SD**
	**(mm)**	**(%)**	**(mm)**	**(%)**	**(mm)**	**(%)**	**(%)**	**(%)**
1d **(25)**	9	84.71	9	84.71	10	82.35	83.92	1.11
1c **(10)**	9	84.71	9	84.71	9	84.71	84.71	0.00
1b **(1)**	8	87.06	8	87.06	7	89.41	87.84	1.11
1a **(0.25)**	9	84.71	9	84.71	8	87.06	85.49	1.11
2 **(50)**	40	11.76	40	11.76	40	11.76	11.76	0.00
2 **(50)** + 1d **(25)**	8	87.06	8	87.06	8	87.06	87.06	0.00
2 **(50)** + 1c **(10)**	6	91.76	2.5	100.00	5	94.12	95.29	3.46
2 **(50)** + 1b **(1)**	7	89.41	6	91.76	7	89.41	90.20	1.11
2 **(50)** + 1a **(0.25)**	5	94.12	5	94.12	6	91.76	93.33	1.11
3 **(25)**	2.5	100.00	2.5	100.00	2.5	100.00	100.00	0.00
3 **(25)** + 1d **(25)**	2.5	100.00	2.5	100.00	2.5	100.00	100.00	0.00
3 **(25)** + 1c **(10)**	2.5	100.00	2.5	100.00	2.5	100.00	100.00	0.00
3 **(25)** + 1b **(1)**	2.5	100.00	2.5	100.00	2.5	100.00	100.00	0.00
3 **(25)** + 1a **(0.25)**	2.5	100.00	2.5	100.00	2.5	100.00	100.00	0.00
4 **(50)**	36	21.18	35	23.53	36	21.18	21.96	1.11
4 **(50)** + 1d **(25)**	7	89.41	6	91.76	7	89.41	90.20	1.11
4 **(50)** + 1c **(10)**	5	94.12	2.5	100.00	2.5	100.00	98.04	2.77
4 **(50)** + 1b **(1)**	2.5	100.00	2.5	100.00	2.5	100.00	100.00	0.00
4 **(50)** + 1a **(0.25)**	9	84.71	10	82.35	10	82.35	83.14	1.11
5 **(25)**	28	40.00	30	35.29	28	40.00	38.43	2.22
5 **(25)** + 1d **(25)**	6	91.76	5	94.12	6	91.76	92.55	1.11
5 **(25)** + 1c **(10)**	3	98.82	2.5	100.00	3	98.82	99.22	0.55
5 **(25)** + 1b **(1)**	5	94.12	3	98.82	5	94.12	95.69	2.22
5 **(25)** + 1a **(0.25)**	7	89.41	6	91.76	4	96.47	92.55	2.93
PDA + 1%DMSO	45	0.00	45	0.00	45	0.00	0.00	0.00

Table S5. Additional inhibition / promotion against *P. infestans, %* (SD: standard deviation)

Substance (concentration in mg/L)	Towards substance alone					Towards tacrolimus				
	**i**	**ii**	**iii**	**Mean**	**SD**	**i**	**ii**	**iii**	**Mean**	**SD**
2 **(50)** + 1d **(25)**	34.56	42.36	28.24	35.05	7.07	12.34	9.41	4.71	8.82	3.85
2 **(50)** + 1c **(10)**	32.10	30.59	23.53	28.74	4.57	12.35	2.35	-2.35	4.12	7.51
2 **(50)** + 1b **(1)**	22.22	37.65	28.24	29.37	7.78	0	11.76	7.06	6.27	5.92
2 **(50)** + 1a **(0.25)**	39.50	42.36	35.29	39.05	3.56	7.40	7.06	7.05	7.17	0.20
3 **(25)** + 1d **(25)**	-2.46	-4.70	-7.06	-4.74	2.30	9.88	0	2.35	4.08	5.16
3 **(25)** + 1c **(10)**	-14.81	-18.82	-21.18	-18.27	3.22	0	-9.41	-14.12	-7.84	7.19
3 **(25)** + 1b **(1)**	-17.28	-30.59	-28.23	-25.37	7.10	-4.94	-18.83	-16.47	-13.41	7.43
3 **(25)** + 1a **(0.25)**	-2.46	-18.82	-11.76	-11.01	8.21	0	-16.47	-7.06	-7.84	8.26
4 **(50)** + 1d **(25)**	22.23	14.12	14.12	16.82	4.68	9.88	0	4.71	4.86	4.94
4 **(50)** + 1c **(10)**	24.69	18.82	18.82	20.78	3.39	14.81	9.41	7.06	10.43	3.97
4 **(50)** + 1b **(1)**	22.23	16.47	11.76	16.82	5.24	9.88	9.41	4.70	8.00	2.86
4 **(50)** + 1a **(0.25)**	9.88	4.70	9.41	8.00	2.86	-12.35	-11.77	-4.71	-9.61	4.25
5 **(25)** + 1d **(25)**	17.29	11.77	30.59	19.88	9.67	9.88	2.35	16.47	9.57	7.07
5 **(25)** + 1c **(10)**	9.88	7.06	18.83	11.92	6.15	4.94	2.35	2.36	3.22	1.49
5 **(25)** + 1b **(1)**	4.94	2.36	18.83	8.71	8.86	-2.47	0	7.06	1.53	4.95
5 **(25)** + 1a **(0.25)**	19.75	7.06	16.47	14.43	6.59	2.46	-4.71	-2.36	-1.54	3.66

Table S6. Additional inhibition / promotion against *F. oxysporum, %* (SD: standard deviation)

Substance (concentration in mg/L)	Towards substance alone					Towards tacrolimus				
	**i**	**ii**	**iii**	**Mean**	**SD**	**i**	**ii**	**iii**	**Mean**	**SD**
2 **(50)** + 1d **(25)**	60.76	45.57	40.00	48.78	10.75	-5.06	-10.13	-16.47	-10.55	5.72
2 **(50)** + 1c **(10)**	63.29	53.17	58.82	58.43	5.07	2.53	-2.53	7.06	2.35	4.80
2 **(50)** + 1b **(1)**	53.16	45.57	49.41	49.38	3.80	-7.60	-7.60	2.35	-4.28	5.74
2 **(50)** + 1a **(0.25)**	50.63	43.04	40.00	44.56	5.47	0	2.53	7.06	3.20	3.58
3 **(25)** + 1d **(25)**	5.06	7.59	11.77	8.14	3.39	5.06	7.59	7.06	6.57	1.33
3 **(25)** + 1c **(10)**	12.66	12.66	18.82	14.71	3.56	17.72	12.66	18.82	16.40	3.29
3 **(25)** + 1b **(1)**	5.06	7.59	4.71	5.79	1.57	10.12	10.12	9.41	9.88	0.41
3 **(25)** + 1a **(0.25)**	0	2.53	9.41	3.98	4.87	15.19	17.72	28.23	20.38	6.91
4 **(50)** + 1d **(25)**	40.51	37.98	32.94	37.14	3.85	2.53	2.53	0	1.69	1.46
4 **(50)** + 1c **(10)**	35.45	35.45	30.58	33.83	2.81	2.53	0	2.35	1.63	1.41
4 **(50)** + 1b **(1)**	25.32	22.79	25.88	24.66	1.65	-7.60	-10.13	2.35	-5.13	6.60
4 **(50)** + 1a **(0.25)**	15.19	15.19	11.76	14.05	1.98	-7.60	-5.07	2.35	-3.44	5.17
5 **(25)** + 1d **(25)**	40.51	40.51	30.59	37.20	5.73	0	2.53	-4.71	-0.73	3.67
5 **(25)** + 1c **(10)**	40.51	40.51	37.65	39.56	1.65	5.06	2.53	7.06	4.88	2.27
5 **(25)** + 1b **(1)**	32.91	27.85	28.24	29.67	2.82	-2.54	-7.60	2.35	-2.60	4.98
5 **(25)** + 1a **(0.25)**	15.19	15.19	9.41	13.26	3.34	-10.13	-7.60	-2.36	-6.70	3.96

Table S7. Additional inhibition / promotion against *C. higginsianum, %* (SD: standard deviation)

Substance (concentration in mg/L)	Towards substance alone					Towards tacrolimus				
	**i**	**ii**	**iii**	**Mean**	**SD**	**i**	**ii**	**iii**	**Mean**	**SD**
2 **(50)** + 1d **(25)**	47.06	49.44	54.12	50.21	3.59	4.71	4.49	2.35	3.85	1.30
2 **(50)** + 1c **(10)**	51.76	53.93	56.47	54.05	2.36	7.05	6.74	4.70	6.16	1.28
2 **(50)** + 1b **(1)**	54.12	53.93	61.18	56.41	4.13	7.06	4.49	9.41	6.99	2.46
2 **(50)** + 1a **(0.25)**	54.12	58.43	61.18	57.91	3.56	7.06	8.99	7.06	7.70	1.11
3 **(25)** + 1d **(25)**	0	0	0	0	0	17.65	16.85	15.29	16.60	1.20
3 **(25)** + 1c **(10)**	0	0	0	0	0	15.29	14.61	15.29	15.06	0.39
3 **(25)** + 1b **(1)**	0	0	0	0	0	12.94	12.36	15.29	13.53	1.55
3 **(25)** + 1a **(0.25)**	0	0	0	0	0	12.94	12.36	12.94	12.75	0.33
4 **(50)** + 1d **(25)**	30.59	24.72	25.88	27.06	3.11	4.71	0	-2.36	0.78	3.60
4 **(50)** + 1c **(10)**	30.59	29.21	25.88	28.56	2.42	2.35	2.25	-2.36	0.75	2.69
4 **(50)** + 1b **(1)**	25.88	24.72	30.59	27.06	3.11	-4.71	-4.49	2.35	-2.28	4.01
4 **(50)** + 1a **(0.25)**	30.59	29.21	28.24	29.35	1.18	0	0	-2.35	-0.78	1.36
5 **(25)** + 1d **(25)**	40.00	38.20	40.00	39.40	1.04	4.71	4.49	2.35	3.85	1.30
5 **(25)** + 1c **(10)**	42.35	40.45	44.70	42.50	2.13	4.70	4.50	7.05	5.42	1.42
5 **(25)** + 1b **(1)**	40.00	42.69	40.00	40.90	1.55	0	4.49	2.35	2.28	2.25
5 **(25)** + 1a **(0.25)**	44.70	42.69	44.70	44.03	1.16	4.70	4.49	4.70	4.63	0.12

Table S8. Additional inhibition / promotion against *A. niger, %* (SD: standard deviation)

Substance (concentration in mg/L)	Towards substance alone					Towards tacrolimus				
	**i**	**ii**	**iii**	**Mean**	**SD**	**i**	**ii**	**iii**	**Mean**	**SD**
2 **(50)** + 1d **(25)**	75.30	75.30	75.30	75.30	0.00	2.35	2.35	4.71	3.14	1.36
2 **(50)** + 1c **(10)**	80.00	88.24	82.36	83.53	4.24	7.05	15.29	9.41	10.58	4.24
2 **(50)** + 1b **(1)**	77.65	80.00	77.65	78.43	1.36	2.35	4.70	0	2.35	2.35
2 **(50)** + 1a **(0.25)**	82.36	82.36	80.00	81.57	1.36	9.41	9.41	4.70	7.84	2.72
3 **(25)** + 1d **(25)**	0	0	0	0	0	15.29	15.29	17.65	16.08	1.36
3 **(25)** + 1c **(10)**	0	0	0	0	0	15.29	15.29	15.29	15.29	0.00
3 **(25)** + 1b **(1)**	0	0	0	0	0	12.94	12.94	10.59	12.16	1.36
3 **(25)** + 1a **(0.25)**	0	0	0	0	0	15.29	15.29	12.94	14.51	1.36
4 **(50)** + 1d **(25)**	68.23	68.23	68.23	68.23	0.00	4.70	7.05	7.06	6.27	1.36
4 **(50)** + 1c **(10)**	72.94	76.47	78.82	76.08	2.96	9.41	15.29	15.29	13.33	3.39
4 **(50)** + 1b **(1)**	78.82	76.47	78.82	78.04	1.36	12.94	12.94	10.59	12.16	1.36
4 **(50)** + 1a **(0.25)**	63.53	58.82	61.17	61.17	2.36	0	-2.36	-4.71	-2.36	2.36
5 **(25)** + 1d **(25)**	51.76	58.83	51.76	54.12	4.08	7.05	9.41	9.41	8.62	1.36
5 **(25)** + 1c **(10)**	58.82	64.71	58.82	60.78	3.40	14.11	15.29	14.11	14.50	0.68
5 **(25)** + 1b **(1)**	54.12	63.53	54.12	57.26	5.43	7.06	11.76	4.71	7.84	3.59
5 **(25)** + 1a **(0.25)**	49.41	56.47	56.47	54.12	4.08	4.70	7.05	9.41	7.05	2.36

Table S9. Spearman's correlations of substances' **1-5** physico-chemical data *versus* their average individual antifungal activities

Average activity, %	Spearman's rho towards	MW	HA	AHA	sp3	RB	HBA	HBD	MR	TPSA	ESOL	Consensus
Aspergillus niger	Correlation Coefficient	.700	.700	.112	.700	.872	.783	.158	.700	.359	-.700	.600
	Sig. (2-tailed)	.188	.188	.858	.188	.054	.118	.800	.188	.553	.188	.285
	N	5	5	5	5	5	5	5	5	5	5	5
Colletotrichum higginsianum	Correlation Coefficient	.500	.500	.112	.500	.872	.783	.158	.500	.359	-.500	.300
	Sig. (2-tailed)	.391	.391	.858	.391	.054	.118	.800	.391	.553	.391	.624
	N	5	5	5	5	5	5	5	5	5	5	5
Phytophthora infestans	Correlation Coefficient	.700	.700	.112	.700	.872	.783	.158	.700	.359	-.700	.600
	Sig. (2-tailed)	.188	.188	.858	.188	.054	.118	.800	.188	.553	.188	.285
	N	5	5	5	5	5	5	5	5	5	5	5
Fusarium oxysporum	Correlation Coefficient	.500	.500	.112	.500	.872	.783	.158	.500	.359	-.500	.300
	Sig. (2-tailed)	.391	.391	.858	.391	.054	.118	.800	.391	.553	.391	.624
	N	5	5	5	5	5	5	5	5	5	5	5

MW - molecular weight, g/mol; HA - number of heavy atoms; AHA - number of aromatic heavy atoms; sp3 - fraction Csp3; RB - number of rotatable bonds; HBA - number of H-bond acceptors; HBD - number of H-bond donors; MR - molar refractivity; TPSA - topological polar surface area, Å². ESOL - water solubility coefficient. Consensus – lipophilicity coefficient.

Table S10. Descriptives of formed bonds to chitin deacetylase of *A. niger* (PDB ID: 7BLY) in order of decreasing affinity

#	Bond from - to	Dist., Å	Category	Type
**5**	:UNL1:H - A:ASP47:OD2	2.98162	HB	Conventional HB
	:UNL1:H - A:ASP48:OD1	2.63156	HB	Conventional HB
	:UNL1:H - A:HIS97:NE2	2.82630	HB	Conventional HB
	:UNL1:H - A:HIS195:NE2	3.06087	HB	Conventional HB
	:UNL1:H - A:HIS101:NE2	2.87160	HB	Conventional HB
	:UNL1:H - A:ASP48:OD2	2.49472	HB	Conventional HB
	A:HIS101 - :UNL1	5.18357	Hydrophobic	Pi-Pi T-shaped
	A:PHE139 - :UNL1	5.08185	Hydrophobic	Pi-Pi T-shaped
	A:TYR138:C,O;PHE139:N - :UNL1	4.57714	Hydrophobic	Amide-Pi Stacked
	A:TYR166 - :UNL1	4.36084	Hydrophobic	Pi-Alkyl
	:UNL1 - A:LEU193	5.05138	Hydrophobic	Pi-Alkyl
**7**	A:ASP162:OD2 - :UNL1	4.77741	Electrostatic	Pi-Anion
	A:TYR138 - :UNL1	5.16376	Hydrophobic	Pi-Pi T-shaped
	A:PHE139 - :UNL1	4.92188	Hydrophobic	Pi-Pi T-shaped
	A:HIS195 - :UNL1	4.30177	Hydrophobic	Pi-Pi T-shaped
	:UNL1 - A:TYR138	5.03862	Hydrophobic	Pi-Pi T-shaped
	A:TYR138:C,O;PHE139:N - :UNL1	4.06381	Hydrophobic	Amide-Pi Stacked
	A:TYR138 - :UNL1:CL	5.19386	Hydrophobic	Pi-Alkyl
	A:PHE139 - :UNL1:C	4.63293	Hydrophobic	Pi-Alkyl
**4**	:UNL1:H - A:ASP47:OD2	2.70330	HB	Conventional HB
	:UNL1:H - A:ASP48:OD1	2.80487	HB	Conventional HB
	:UNL1:H - A:HIS195:NE2	2.83636	HB	Conventional HB
	:UNL1:H - A:ASP48:OD1	2.78882	HB	Conventional HB
	:UNL1:H - A:HIS101:NE2	2.85082	HB	Conventional HB
	:UNL1:H - A:ASP48:OD2	2.54247	HB	Conventional HB
	:UNL1:H - A:HIS101:NE2	3.05610	HB	Conventional HB
	A:LEU193:CD2 - :UNL1	3.93587	Hydrophobic	Pi-Sigma
	A:TYR138 - :UNL1	5.76257	Hydrophobic	Pi-Pi T-shaped
	A:PHE139 - :UNL1	4.88005	Hydrophobic	Pi-Pi T-shaped
	:UNL1 - A:TYR138	4.93936	Hydrophobic	Pi-Pi T-shaped
	A:TYR138:C,O;PHE139:N - :UNL1	4.50182	Hydrophobic	Amide-Pi Stacked
	A:TYR166 - :UNL1	4.56746	Hydrophobic	Pi-Alkyl
**3**	:UNL1:C - A:HIS101:NE2	3.60228	HB	Carbon HB
	A:TYR166 - :UNL1	3.67595	Hydrophobic	Pi-Pi Stacked
	A:TYR138 - :UNL1	5.85059	Hydrophobic	Pi-Pi T-shaped
	A:HIS195 - :UNL1	5.25469	Hydrophobic	Pi-Pi T-shaped
	:UNL1 - A:PHE139	5.12894	Hydrophobic	Pi-Pi T-shaped
	:UNL1 - A:LEU193	5.16708	Hydrophobic	Pi-Alkyl
**6**	A:LYS164:NZ - A:UNK1:O	3.21216	HB	Conventional HB
	A:UNK1:H - A:ASP162:OD2	2.23064	HB	Conventional HB
	A:UNK1:H - A:HIS195:NE2	2.88467	HB	Conventional HB
	A:UNK1:H - A:ASP48:OD2	2.67908	HB	Conventional HB
	A:UNK2:H - A:TYR166	3.28050	HB	Pi-Donor HB
	A:TYR166 - A:UNK2:C	5.07634	Hydrophobic	Pi-Alkyl
**1**	A:TYR138 - :UNL1:C	5.15402	Hydrophobic	Pi-Alkyl
	A:TYR138 - :UNL1:C	4.46736	Hydrophobic	Pi-Alkyl
	A:TYR166 - :UNL1	3.87159	Hydrophobic	Pi-Alkyl
**2**	:UNL1:H - A:ASP47:OD2	2.46783	HB	Conventional HB
	:UNL1:C - A:PHE139	3.59347	Hydrophobic	Pi-Sigma
	:UNL1:C - A:LEU193	4.98336	Hydrophobic	Alkyl

Table S11. Descriptives of formed bonds to chitin deacetylase of *A. nidulans* (PDB ID: 2Y8U) in order of decreasing affinity

#	Bond from - to	Dist., Å	Category	Type
5	:UNL1:H - B:ASP47:OD2	2.80089	HB	Conventional HB
	:UNL1:H - B:ASP48:OD1	2.72298	HB	Conventional HB
	:UNL1:H - B:HIS97:NE2	2.57584	HB	Conventional HB
	:UNL1:H - B:ASP48:OD2	2.85985	HB	Conventional HB
	:UNL1:H - B:ASP48:OD2	2.22686	HB	Conventional HB
	:UNL1 - B:TYR166	4.91399	Hydrophobic	Pi-Pi Stacked
	B:HIS101 - :UNL1	4.96149	Hydrophobic	Pi-Pi T-shaped
	A:TYR138 - :UNL1:C	4.96885	Hydrophobic	Pi-Alkyl
	A:TYR166 - :UNL1	4.84305	Hydrophobic	Pi-Alkyl
	B:TYR166 - :UNL1:C	4.82433	Hydrophobic	Pi-Alkyl
	:UNL1 - B:LEU139	5.21146	Hydrophobic	Pi-Alkyl
	:UNL1 - B:LEU194	5.25501	Hydrophobic	Pi-Alkyl
7	A:TYR166:OH - :UNL1:O	3.11380	HB	Conventional HB
	B:TYR166:OH - :UNL1:N	2.83306	HB	Conventional HB
	B:HIS196:CE1 - :UNL1:O	3.71522	HB	Carbon HB
	:UNL1:C - B:ASP48:OD2	3.69202	HB	Carbon HB
	A:TYR166:OH - :UNL1	4.07738	HB	Pi-Donor HB
	A:TYR166 - :UNL1	4.11548	Hydrophobic	Pi-Pi Stacked
	B:TYR166 - :UNL1	5.03911	Hydrophobic	Pi-Pi T-shaped
	B:HIS196 - :UNL1	4.31393	Hydrophobic	Pi-Pi T-shaped
	:UNL1 - B:TYR138	5.12809	Hydrophobic	Pi-Pi T-shaped
	:UNL1:CL - B:LEU139	4.22426	Hydrophobic	Alkyl
	:UNL1:CL - B:LEU194	4.68699	Hydrophobic	Alkyl
	A:HIS101 - :UNL1:C	5.36555	Hydrophobic	Pi-Alkyl
	A:TYR138 - :UNL1:C	4.97760	Hydrophobic	Pi-Alkyl
4	:UNL1:H - B:ASP47:OD2	2.98211	HB	Conventional HB
	:UNL1:H - B:ASP48:OD1	2.78720	HB	Conventional HB
	:UNL1:H - B:HIS97:NE2	2.11523	HB	Conventional HB
	:UNL1:H - B:HIS101:NE2	3.06540	HB	Conventional HB
	:UNL1:H - B:ASP48:OD2	2.35145	HB	Conventional HB
	:UNL1 - B:TYR166	4.99064	Hydrophobic	Pi-Pi Stacked
	:UNL1 - B:TYR138	5.01241	Hydrophobic	Pi-Pi T-shaped
	A:TYR166 - :UNL1	5.42975	Hydrophobic	Pi-Alkyl
	B:TYR166 - :UNL1	4.73867	Hydrophobic	Pi-Alkyl
	:UNL1 - B:LEU139	5.02651	Hydrophobic	Pi-Alkyl
	:UNL1 - B:LEU194	5.05554	Hydrophobic	Pi-Alkyl
3	:UNL1:H - B:HIS196:NE2	2.58544	HB	Conventional HB
	:UNL1:C - B:HIS101:NE2	3.56747	HB	Carbon HB
	B:TYR166 - :UNL1	3.79118	Hydrophobic	Pi-Pi Stacked
	B:TYR138 - :UNL1	5.45556	Hydrophobic	Pi-Pi T-shaped
	B:HIS196 - :UNL1	5.45597	Hydrophobic	Pi-Pi T-shaped
	B:TYR138:C,O;LEU139:N - :UNL1	4.47648	Hydrophobic	Amide-Pi Stacked
	B:TYR138 - :UNL1	5.37462	Hydrophobic	Pi-Alkyl
	:UNL1 - B:LEU139	4.88582	Hydrophobic	Pi-Alkyl
	:UNL1 - B:LEU194	5.17241	Hydrophobic	Pi-Alkyl
6	A:GLN169:NE2 - A:UNK2:O	2.85033	HB	Conventional HB
	A:TYR200:N - A:UNK2:O	3.37169	HB	Conventional HB
	A:TYR200:N - A:UNK2:O	3.05134	HB	Conventional HB
	B:ASP75:N - A:UNK2:O	3.05606	HB	Conventional HB
	B:HIS101:ND1 - A:UNK1:O	2.91237	HB	Conventional HB
	B:TYR138:OH - A:UNK1:O	2.97936	HB	Conventional HB
	A:UNK2:H - B:TYR99:O	2.83443	HB	Conventional HB
	A:UNK1:H - B:ASP48:OD2	2.16910	HB	Conventional HB
	A:UNK1:H - A:TYR166:O	2.80140	HB	Conventional HB
	A:UNK1:H - B:TYR138:OH	2.12115	HB	Conventional HB
	A:UNK1:H - B:ASP100:O	2.72129	HB	Conventional HB
	A:TRP201:CD1 - A:UNK2:O	3.50056	HB	Carbon HB
	A:UNK2:C - A:UNK1:O	3.26472	HB	Carbon HB
	A:UNK2:C - B:ASP100:OD1	3.67098	HB	Carbon HB
	A:UNK1:H - B:HIS101	2.40928	HB	Pi-Donor HB
	A:UNK2 - B:LEU73	5.46725	Hydrophobic	Pi-Alkyl
1	:UNL1:H - B:ASP100:O	2.22614	HB	Conventional HB
	A:HIS199:CE1 - :UNL1:O	2.98483	HB	Carbon HB
	:UNL1:C - :UNL1:O	3.16631	HB	Carbon HB
	:UNL1:C - B:HIS101	3.62918	Hydrophobic	Pi-Sigma
	:UNL1 - A:ILE198	5.16828	Hydrophobic	Alkyl
	:UNL1:C - A:ILE198	4.57220	Hydrophobic	Alkyl
	A:HIS101 - :UNL1:C	4.25195	Hydrophobic	Pi-Alkyl
	A:TYR138 - :UNL1:C	4.78218	Hydrophobic	Pi-Alkyl
	A:TYR138 - :UNL1:C	5.03270	Hydrophobic	Pi-Alkyl
	A:TYR166 - :UNL1:C	4.85782	Hydrophobic	Pi-Alkyl
	A:TYR166 - :UNL1:C	5.11688	Hydrophobic	Pi-Alkyl
	A:HIS199 - :UNL1:C	4.22824	Hydrophobic	Pi-Alkyl
	A:HIS199 - :UNL1	5.16460	Hydrophobic	Pi-Alkyl
	B:TYR166 - :UNL1:C	4.63109	Hydrophobic	Pi-Alkyl
	B:TYR166 - :UNL1:C	5.33172	Hydrophobic	Pi-Alkyl
	B:HIS199 - :UNL1:C	4.57750	Hydrophobic	Pi-Alkyl

**2**	B:TYR138:N - :UNL1:O	3.12194	HB	Conventional HB
	:UNL1:H - B:ASP48:OD1	2.45275	HB	Conventional HB
	:UNL1:H - B:HIS101:NE2	2.24623	HB	Conventional HB
	:UNL1:C - B:TYR166	3.74718	Hydrophobic	Pi-Sigma
	B:TYR138 - :UNL1:C	5.41309	Hydrophobic	Pi-Alkyl
	B:HIS196 - :UNL1:C	4.99139	Hydrophobic	Pi-Alkyl
